# COL4A1‐Related Leukoencephalopathy and Microangiopathy: A Case Series of Two Palestinian Siblings

**DOI:** 10.1002/ccr3.70964

**Published:** 2025-09-24

**Authors:** Thkra Meshal, Amal M. Shawabka, Kareem lbraheem, Rosol Iwaiwi, Nissreen E. Thafer, Jinan H. Afana, Ahmad G. Hammouri, Osama Atawneh

**Affiliations:** ^1^ Faculty of Medicine Palestine Polytechnic University Hebron Palestine; ^2^ Palestinian Clinical Research Center Bethlehem Palestine; ^3^ Radiology Department Al‐Ahli Hospital Hebron Palestine; ^4^ Pediatrics, Pediatric Medicine Palestine Red Crescent Society (PRCS) Hospital Hebron Palestine

**Keywords:** case report, cerebral small vessel disease, COL4A1 gene, leukoencephalopathy, microangiopathy, pediatrics neurology

## Abstract

We report two Palestinian siblings with a pathogenic COL4A1 mutation, presenting with congenital cataracts, seizures, developmental delay, and antenatal intracerebral hemorrhages. Despite sharing the same genetic variant, they exhibited striking phenotypic variability. This case underscores the importance of recognizing COL4A1‐related manifestations—including neurological and ophthalmological features—for timely diagnosis and genetic counseling in familial small vessel disease.

## 1 Introduction

Cerebral small vessel disease (cSVD) is a set of diseases affecting the structure and function of the small arteries responsible for supplying blood to the brain and is a major contributor to stroke and cognitive impairment [1]. Detecting these dysfunctional vessels is challenging using conventional imaging techniques due to their small size and the marked variability in clinical manifestations, which does not make the diagnosis any easier. However, recent advances in diagnostic methods have led to an increase in the identification of new cases [1, 2]. While the majority of cases are sporadic and associated with multifactorial risk factors, an increasing number of hereditary monogenic mutations are being identified [1].

A recently recognized monogenic cause of cSVD is *COL4A1* mutations, most of them being missense, resulting in the substitution of a glycine with a different amino acid [3, 4].

*COL4A1* is responsible for encoding the alpha‐1 chain of type IV collagen, which is a crucial component of the vascular basement membrane providing mechanical support [5].

These mutations lead to the synthesis of structurally abnormal proteins, increasing the fragility of the vessel wall, causing a systemic vascular basement membrane disease in multiple organ systems, with a major site of damage occurring in the brain [3].

Characterized by variable phenotypic expression in children, including congenital porencephaly, intracerebral hemorrhage, and infantile hemiparesis; neurological features such as stroke, migraine, and epilepsy; and systemic features including ocular, renal, and muscular involvement [6].

Early identification of patients carrying *COL4A1* mutations has important clinical consequences due to its diagnostic challenges, variable manifestations with overlapping systemic and central nervous system features leading to severe neurologic conditions. This report details two Palestinian siblings with COL4A1 variant mutation: one with antenatal brain injury and seizures, the other with delayed hydrocephalus and epilepsy. These divergent outcomes highlight interactions between genetic, environmental, and prenatal factors.

## 2 Case Presentation

### 2.1 Case One

#### 2.1.1 Case History and Examination

We present the case of a 5‐year‐old male, initially diagnosed at 3 days old after presenting to the emergency room with abnormal, rhythmic, multifocal myoclonic movements in his upper and lower limbs. These movements lasted 1 min and were not accompanied by eye‐rolling, cyanosis, or impaired awareness. He experienced delayed walking until 24 months, but was otherwise normal. There was no history of fever, poor feeding, hypoactivity, or trauma.

On examination, his weight was 2660 g (< 3rd percentile), length 47 cm (10th percentile), and head circumference 32 cm (< 3rd percentile). Mild lower limb spasticity with brisk reflexes but preserved strength was noted. His vital signs and blood gas analysis were normal. An echocardiogram was performed to rule out intracardiac thrombosis and was normal except for a patent foramen ovale (PFO). The patient's parents are consanguineous (first cousins). The mother is healthy, while the father has a history of encephalomalacia with intellectual disability, bilateral cataracts, and abnormal gait.

#### 2.1.2 Methods (Differential Diagnosis, Investigations, and Treatment)

**FIGURE 1:**
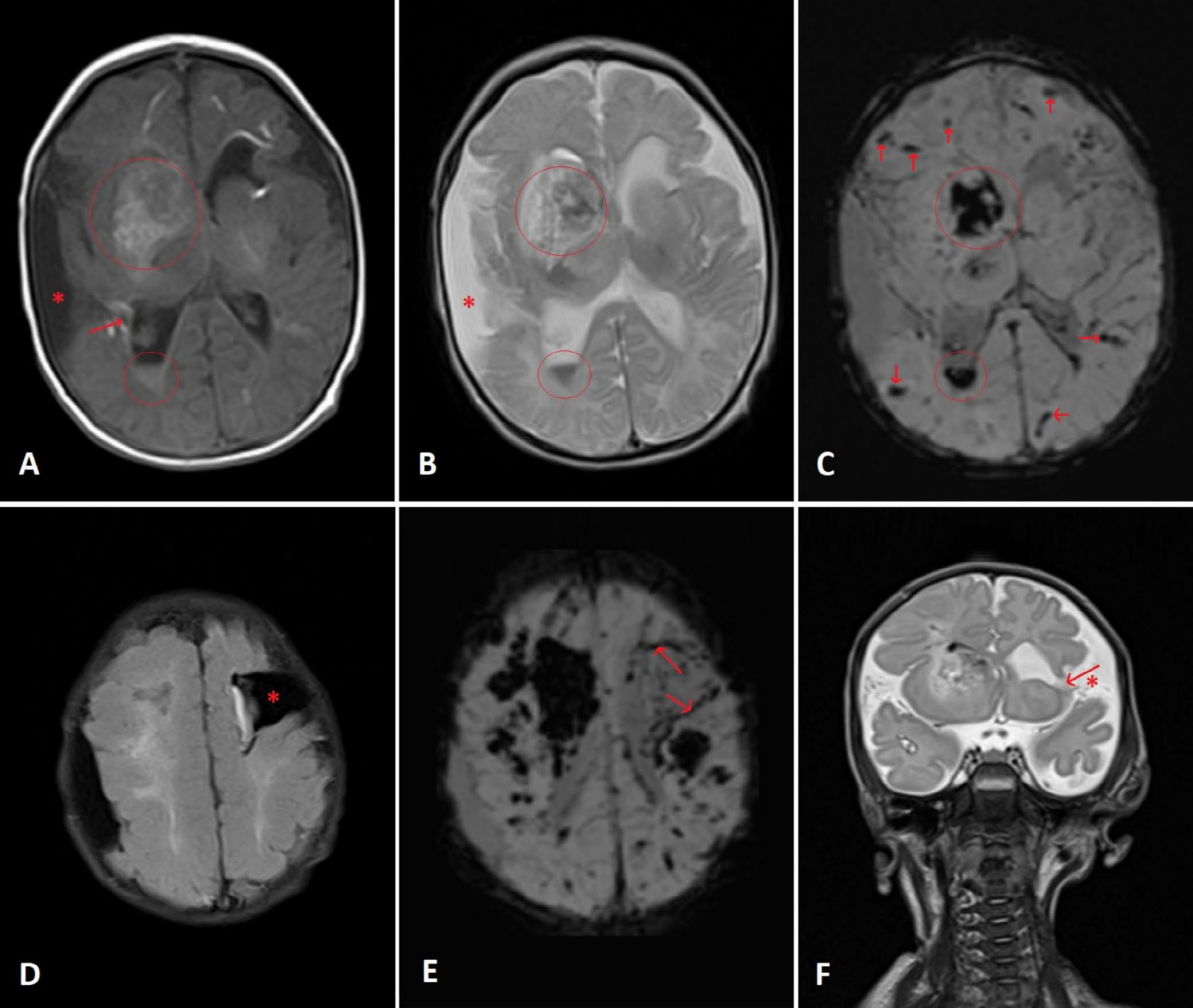
Multiplanar multisequence magnetic resonance imaging for patient 1's brain (A: axial T1WI, B: axial T2WI, C: axial susceptibility‐weighted images (SWI), D: axial fluid‐attenuated inversion recovery (FLAIR) image, E: axial susceptibility‐weighted images (SWI), F: coronal T2WI). The images show intraventricular hemorrhage mainly at the right lateral ventricle with intraparenchymal involvement of the right frontal periventricular area characterized by hyperintense T1 signal intensity (SI) (circles in A), hypointense T2 SI (circles in B) with blooming artifact at the SWI (circles in C), suggesting subacute hemorrhages. Other foci of blooming artifact are shown, representing microhemorrhages (arrows in C). An area of encephalomalacia is noted in the left fronto‐parietal region (asterisk in D & F), reaching the left lateral ventricle (arrow in F) as well as a surrounding rim of blooming artifact (arrows in E), suggesting superficial hemosiderosis. Another area of extra‐axial collection and encephalomalacia is seen in the right parietal region (asterisk in A & B) reaching the posterior horn of the right lateral ventricle (arrow in A). Findings mostly represent sequelae related to antenatal insults—likely hemorrhages.

**FIGURE 2:**
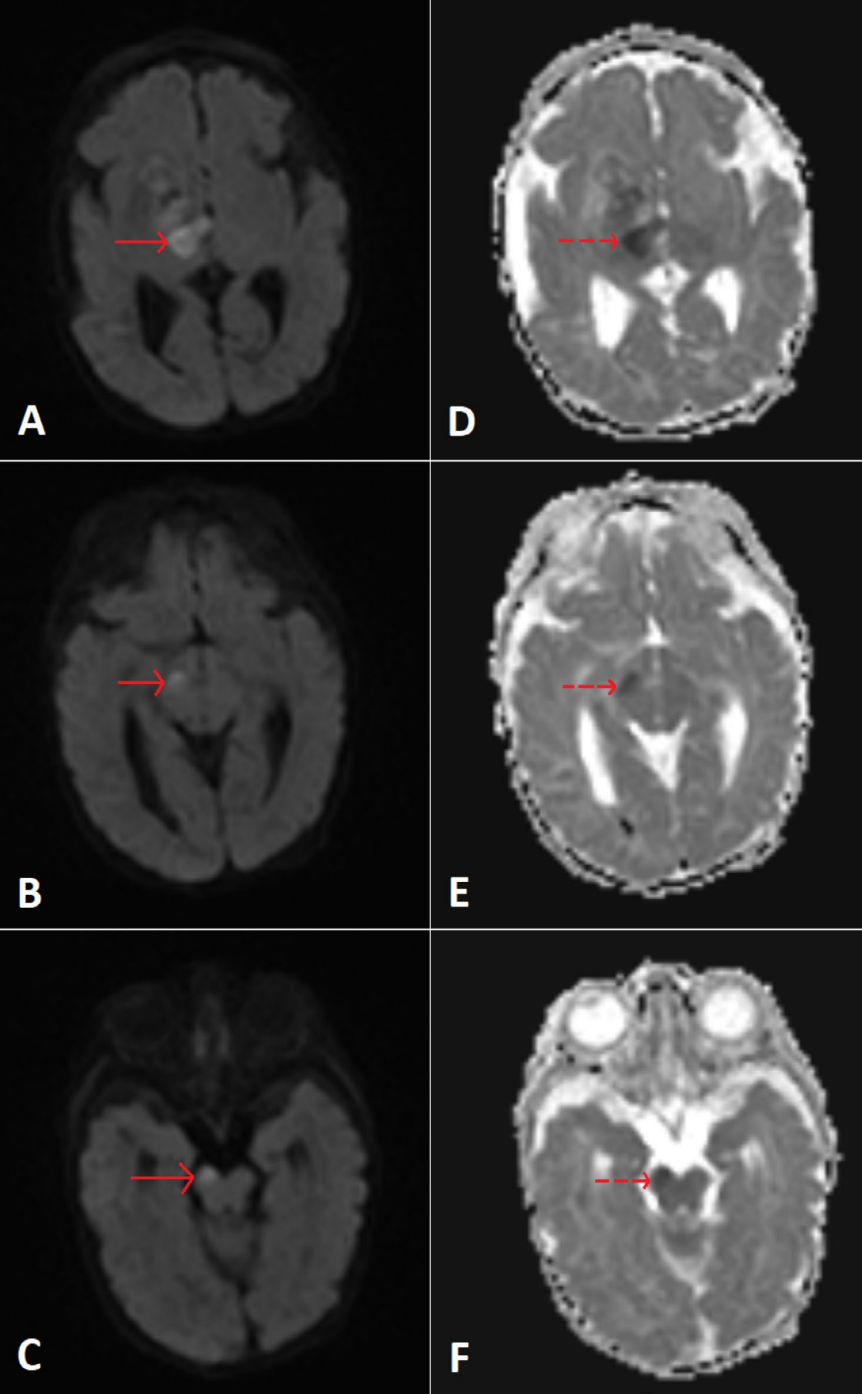
Axial diffusion‐weighted images for patient 1's brain (A, B & C: diffusion‐weighted images (DWI), D, E & F: apparent diffusion coefficient (ADC)). The images are noted showing true diffusion restriction (high DWI—red arrows—with corresponding low ADC value—dashed red arrows) involving the right subthalamic nucleus, the right anterior aspect of the mid‐brain and pons (right corticospinal tract), mostly representing Wallerian degeneration.

An EEG showed an abnormal record with low‐voltage activity over the left side and bilateral focal epileptogenic activity. MRI of the brain revealed multiple foci of intracerebral hemorrhages, secondary to strokes. Additionally, there were areas of encephalomalacia in the left frontoparietal region with gliotic changes (Figure 1), related to hemorrhagic antenatal insults, as well as features of right‐sided Wallerian degeneration (Figure 2). MRA and MRV were normal. The infant was diagnosed with spastic cerebral palsy associated with neonatal seizures and strokes. He was started on phenobarbital 7.5 mg twice daily. The family was advised to undergo thrombophilia, ophthalmic, and hearing evaluations.

**FIGURE 3:**
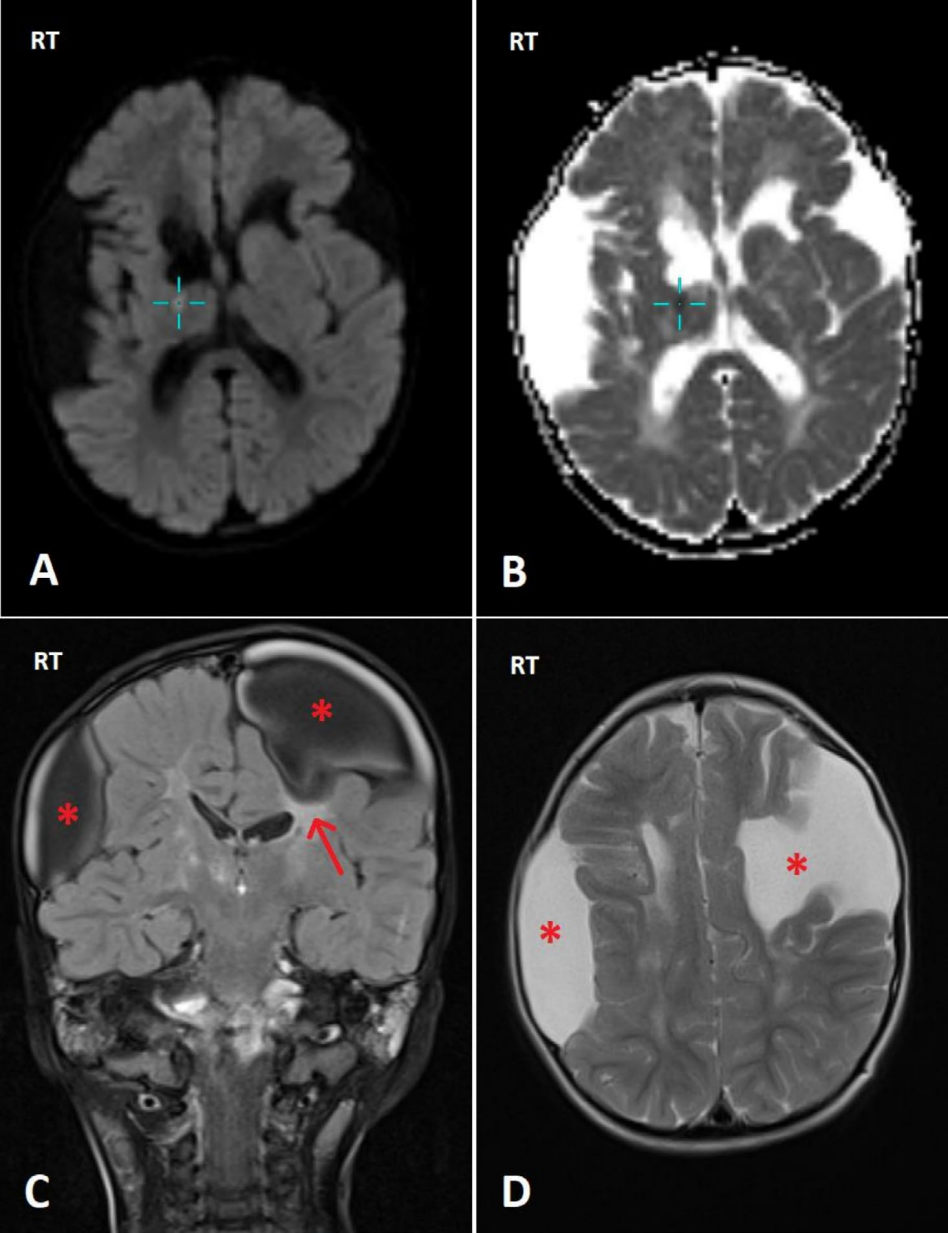
Multiplanar multisequence magnetic resonance imaging for patient 1's brain done 3 years later (A: diffusion‐weighted images (DWI), B: apparent diffusion coefficient (ADC), C: coronal fluid‐attenuated inversion recovery (FLAIR) image, D: axial T2WI). The images show a new acute lacuna infarct of about 5 mm, causing true diffusion restriction seen at the right internal capsule/thalamus (cursor in A and B). The previously seen right extra‐axial lentiform‐shaped collection as well as the left fronto‐parietal collection are also noted showing CSF density without definite communication with the ventricular system (asterisk in C & D). You can notice the lack of cortical covering at the left collection with the surrounding area of gliosis manifested as FLAIR high signal intensity (arrow in C), likely suggesting porencephalic cysts related to old intracranial hemorrhage.

The thrombophilia test was negative, the audiology test was normal, and the ophthalmic evaluation revealed bilateral cataracts, which were corrected by secondary intraocular lens implantation in the right and left eyes at the age of 4. Physiotherapy, occupational therapy, hydrotherapy, and physical rehabilitation were recommended as part of the treatment plan. Genetic testing confirmed a COL4A1 missense mutation (c.2707G > C, p.Gly903Arg). Interictal examination reveals global developmental delay (delayed walking until 24 months, limited speech to short phrases), wide‐based ataxic gait, mild lower limb spasticity with brisk reflexes but preserved strength, and dysmetria on finger‐nose testing. A follow‐up brain MRI was performed at the age of 3 years and demonstrated porencephalic cyst formation secondary to old cerebrovascular insults (Figure 3).

#### 2.1.3 Conclusions and Results (Outcome and Follow‐Up)

The child was diagnosed with spastic cerebral palsy, neonatal strokes, and congenital cataracts, requiring multidisciplinary rehabilitation. The genetic confirmation of PADMAL provided clarity on the underlying cause and facilitated family counseling. Follow‐up imaging further confirmed the progressive nature of cerebrovascular changes, highlighting the importance of early recognition and intervention in such cases.

### 2.2 Case Two

#### 2.2.1 Case History and Examination

We present a case of a 3‐year‐old male, the sibling of the child described in case 1, who was prenatally diagnosed with neurological and ophthalmological abnormalities. A detailed prenatal ultrasound revealed a hypoechoic lesion suggestive of intraventricular hemorrhage and periventricular calcifications in the left ventricle. A subsequent fetal MRI showed ventriculomegaly, suspected posthemorrhagic hydrocephalus, encephalomalacia, and possible agenesis of the corpus callosum (image not available).

**FIGURE 4:**
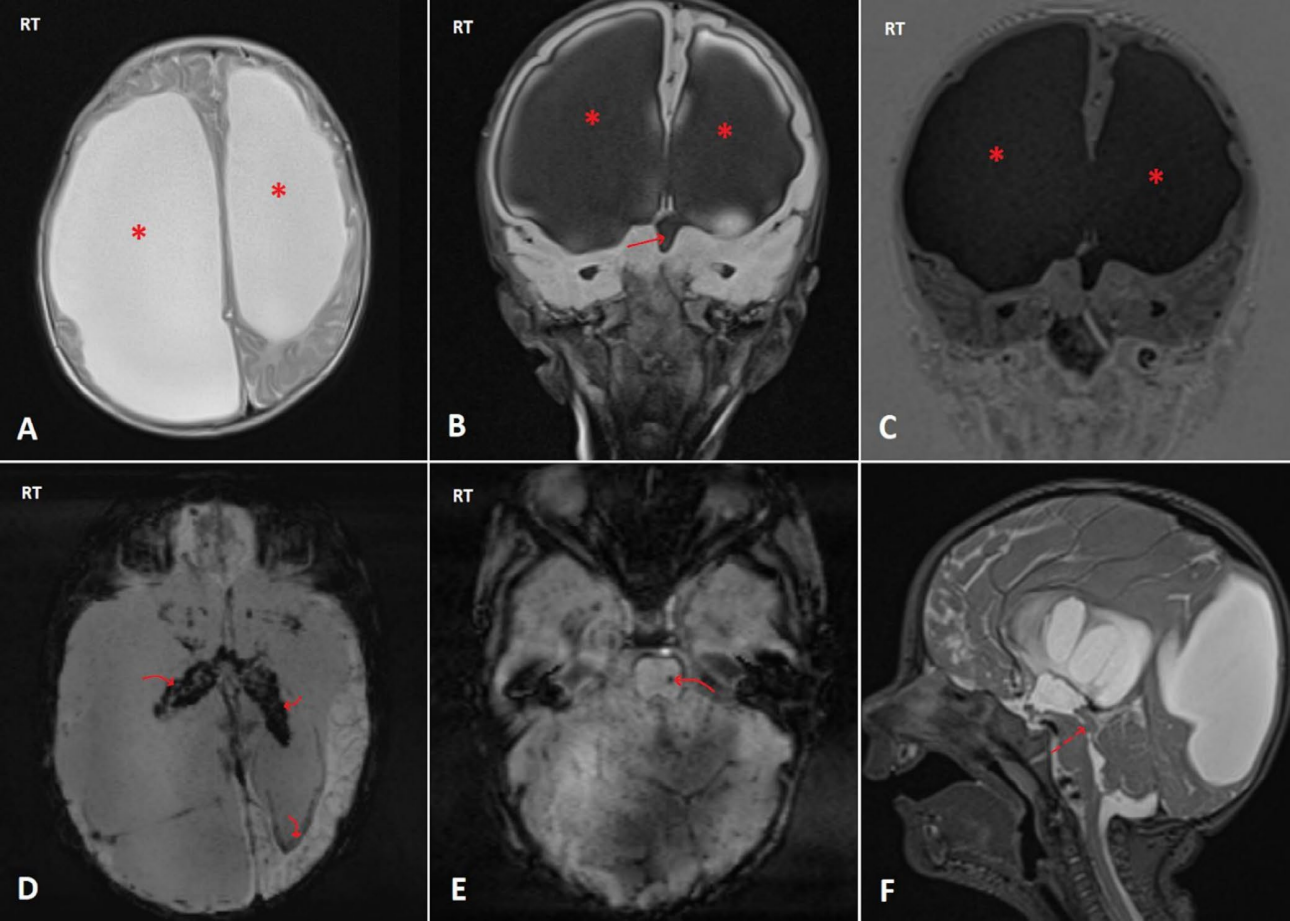
Multiplanar multisequence magnetic resonance imaging for patient 1's brain (A: axial T2WI, B: coronal fluid‐attenuated inversion recovery (FLAIR) image, C: coronal T1 inversion recovery pulse sequence, D&E: axial susceptibility‐weighted images (SWI), F: midline sagittal T2W image). The images show severe asymmetric dilatation of both lateral ventricles, more at the right side (asterisk in A, B, & C) as well as the third ventricle (solid arrow in B), with subsequent white matter volume loss, in addition to significant blooming artifact observed inside both lateral ventricles at the SWI images (curved arrows in D). Sagittal T2W images revealed a small (about 2.5 mm) hypointense component (dashed arrow in F) seen inside the cerebral aqueduct of Sylvius with a nondilated fourth ventricle. Findings are likely related to obstructive hydrocephalus related to antenatal/chronic intraventricular hemorrhage. Another tiny focus of blooming artifacts is observed in the left pons at SWI images (curved arrow in E), suggesting microhemorrhage.

Due to these findings, he was delivered via cesarean section at 38 + 3 weeks of gestation, with a birth weight of 2900 g (50th percentile) and a head circumference of 38 cm (> 97th percentile). Postnatal examination identified bilateral congenital cataracts, which were corrected by lens replacement surgery at 6 months. At 4 months, he exhibited macrocephaly (head circumference 43.5 cm, > 97th percentile) and developmental delays, including marked head lag, axial hypotonia, clonus, downward gaze, and bilateral hydrocele. A brain MRI confirmed severe ventricular dilation and partial agenesis of the septum pellucidum (Figure 4).

#### 2.2.2 Methods (Differential Diagnosis, Investigations, and Treatment)

At 7 months, he developed exophthalmos, exaggerated deep tendon reflexes, and a bulging anterior fontanelle, raising concern for increased intracranial pressure, leading to ventriculoperitoneal (VP) shunt placement. By 9 months, he experienced status epilepticus, requiring hospitalization and initiation of anticonvulsants. An EEG showed diffuse high‐voltage slowing intermixed with multifocal sharp‐wave activity, predominantly on the right side, consistent with a cortical epileptic focus. A similar event recurred at 2 years old. Genetic testing confirmed a COL4A1 mutation, the same as his brother (Case 1) (Table 1).

**TABLE 1:** Comparative clinical summary.

Feature	Case 1 (5 y.o male)	Case 2 (3 y.o male)
Genetic Mutation	COL4A1 (3′ UTR variant)	COL4A1 (3′ UTR variant)
Prenatal Findings	None documented	Intraventricular hemorrhage, ventriculomegaly
Age at Symptom Onset	3 days (neonatal seizures)	Prenatal (ultrasound anomalies)
Key Neurological Issues	Neonatal seizures, spastic CP	Hydrocephalus, status epilepticus
Neuroimaging	Antenatal hemorrhages, encephalomalacia, Wallerian degeneration (Figures 1 and 2)	Posthemorrhagic hydrocephalus, periventricular calcifications (Figure 4)
Ocular Manifestations	Bilateral cataracts (surgically corrected)	Bilateral cataracts (surgically corrected)
Management	Phenobarbital, rehabilitation	VP shunt, anticonvulsants

#### 2.2.3 Conclusions and Results (Outcome and Follow‐Up)

The child was diagnosed with severe neurological impairment, congenital cataracts, and hydrocephalus requiring VP shunting. Despite early seizure control and surgical intervention, he continued to have developmental delays and recurrent seizures, highlighting the progressive nature of COL4A1. The genetic confirmation enabled family counseling and long‐term multidisciplinary management.

## 3 Discussion

COL4A1‐related vasculopathies mutations are autosomal dominant disorders and are associated with a range of cerebral small vessel diseases due to their effect on basement membrane integrity. The *COL4A1* gene, located on chromosome 13q34, encodes the alpha‐1 chain of type IV collagen, which is a crucial structural element of basement membranes, particularly in maintaining the stability of the cerebral microvasculature [3].

These mutations, involving glycine substitutions within the collagenous domain, disrupt the formation of the type IV collagen triple helix, leading to impaired proper secretion and incorporation of collagen into the vascular basement membrane, resulting in structural fragility of small cerebral vessels. Consequently, individuals carrying these mutations are predisposed to a wide range of cerebrovascular manifestations, including perinatal intracerebral hemorrhage, porencephaly, recurrent adult‐onset intracerebral hemorrhages, microbleeds, lacunar strokes, and leukoaraiosis [3].

The impaired vascular integrity, also resulting in endothelial dysfunction, causes blood–brain barrier (BBB) leakage of plasma proteins and other harmful substances into the brain parenchyma, and microangiopathic damage. Also, the accumulation of misfolded proteins within the endoplasmic reticulum (ER) triggers ER stress, impairing cellular function and activating apoptotic pathways, which contribute to tissue damage. These pathological changes play a significant role in the progressive neurological decline observed in individuals with COL4A1 mutations [7, 8].

In addition to affecting the central nervous system, COL4A1‐related mutations are associated with multisystem involvement, owing to the role of type IV collagen in maintaining basement membrane integrity across various organ systems. As a result, these mutations can lead to ocular abnormalities such as cataracts and anterior segment dysgenesis, renal involvement including hematuria or nephropathy, and myopathy affecting the muscular system [8].

Perinatal brain hemorrhages in fetuses and newborns were the first manifestation of COL4A1 mutations to be described [9]. It was demonstrated that brain hemorrhages that occurred during pregnancy or the perinatal period were likewise linked to porencephalic cysts in children with mutations. The deep periventricular brain areas were typically affected by these cysts [10].

Other brain abnormalities associated with prenatal hemorrhages, including schizencephaly, hydranencephaly, hydrocephalus, ventricular asymmetry, periventricular leukomalacia, and brain calcifications, have been documented in mutant offspring in addition to porencephaly [11]. Cortical developmental malformations have also been described as focal cortical dysplasia [12]. The clinical spectrum includes the onset of infantile hemiparesis, spastic tetraparesis, epilepsy, or psychomotor delay of varying degrees, occasionally in conjunction with macrocephaly or microcephaly [10, 11]. Milder symptoms, like isolated minor intellectual disability or soft motor signs, may be displayed by some children. When a late miscarriage occurs or an ultrasound examination reveals a large porencephalic cavity during pregnancy, the diagnosis can be made in the most severe cases [13].

When it comes to the brain, the most commonly affected areas in infants and early children with COL4A1 or COL4A2 mutations are the eyes. The following describes developmental anomalies that affect either the front section of the eye (congenital or juvenile cataract, microcornea, Axenfeld Rieger syndrome), the posterior segment (congenital or juvenile glaucoma, optic nerve dysgenesis), or both. Congenital blindness and microphthalmia are potential outcomes in severe situations. Strabismus occurs often [12, 14]. In both cases presented here, bilateral congenital cataracts were a prominent feature, requiring surgical correction early in life.

The prognosis for patients with COL4A1 mutations varies widely, ranging from severe cases with porencephalic cysts, marked developmental delay, intellectual and motor disturbances, and microcephaly, as seen in Case 1, where the infant exhibited encephalomalacia, Wallerian degeneration, and neonatal seizures. In contrast, milder forms may present with epilepsy as the predominant symptom, accompanied by subtle motor problems, as observed in Case 2, where the child experienced status epilepticus and developmental delays. These milder cases are often misdiagnosed due to nonspecific brain MRI features and are challenging to recognize, especially when epilepsy is the primary symptom. There are many prognostic factors that play a role in the severity of the phenotypic features for those patients, including the age of onset, with earlier onset associated with more severe outcomes, and the presence of structural brain abnormalities such as porencephaly or cortical dysplasia. Also, some reports are indicating that maternal inheritance may increase disease severity due to fetal vulnerability to intrauterine stressors. Furthermore, the co‐occurrence of cerebral microbleeds and vascular damage can lead to such consequences as ischemic stroke or intracerebral hemorrhage [15].

The management of COL4A1 mutation patients involves a comprehensive approach, including detailed clinical assessments and molecular testing for COL4A1, especially in cases of unexplained brain hemorrhage, porencephaly, or eye abnormalities [16]. Currently, there is no specific treatment for those patients. In this disease, patients require careful neurological monitoring, including blood pressure management to reduce stroke risk and antiepileptics for seizures if present. Renal function should be regularly assessed, with ACE inhibitors/ARBs considered for proteinuria. Ophthalmologic evaluations are essential for detecting ocular abnormalities. Muscle cramps may respond to quinine or magnesium. Also, intravenous thrombolysis and extreme physical activities that could be risky for head trauma are not recommended [17]. Genetic counseling is crucial for informing at‐risk family members and considering prenatal diagnosis in future pregnancies [16]. In Case 2, the prenatal diagnosis of intraventricular hemorrhage and periventricular calcifications on detailed ultrasound, along with the family history of COL4A1 mutation, prompted early genetic testing and a multidisciplinary approach to care, highlighting the importance of early intervention and family counseling.

## Author Contributions

**Thkra Meshal:** project administration, resources, software, validation, visualization, writing – original draft, writing – review and editing. **Amal M. Shawabka:** supervision, writing – review and editing. **Kareem lbraheem:** supervision, writing – review and editing. **Rosol Iwaiwi:** supervision, validation, writing – review and editing. **Nissreen E. Thafer:** supervision, writing – review and editing. **Jinan H. Afana:** validation, writing – review and editing. **Ahmad G. Hammouri:** conceptualization, data curation, formal analysis, investigation, methodology. **Osama Atawneh:** conceptualization, supervision, writing – review and editing.

## Consent

Written informed consent was obtained from the patient's parents for the publication of this case series.

## Conflicts of Interest

The authors declare no conflicts of interest.

## Data Availability

The data used to support the findings of this study are included in the article.

## References

[1] S. Guey and H. Chabriat , “Chapter 16 – Monogenic Causes of Cerebral Small Vessel Disease and Stroke,” in Handbook of Clinical Neurology, vol. 204, ed. D. S. Lynch and H. Houlden (Elsevier, 2024), 273–287, https://www.sciencedirect.com/science/article/pii/B9780323992091000181. 10.1016/B978-0-323-99209-1.00018-1 39322384

[2] C. Bordes , M. Sargurupremraj , A. Mishra , and S. Debette , “Genetics of Common Cerebral Small Vessel Disease,” Nature Reviews Neurology 18, no. 2 (2022): 84–101, 10.1038/s41582-021-00592-8. 34987231

[3] I. Volonghi , A. Pezzini , E. Del Zotto , et al., “Role of COL4A1 in Basement‐Membrane Integrity and Cerebral Small‐Vessel Disease. The COL4A1 Stroke Syndrome,” Current Medicinal Chemistry 17, no. 13 (2010): 1317–1324. 20166936 10.2174/092986710790936293

[4] S. Zagaglia , C. Selch , J. R. Nisevic , et al., “Neurologic Phenotypes Associated With COL4A1/2 Mutations: Expanding the Spectrum of Disease,” Neurology 91, no. 22 (2018): e2078–e2088. 30413629 10.1212/WNL.0000000000006567 PMC6282239

[5] S. John , L. Jehi , E. M. Manno , D. S. Conway , and K. Uchino , “COL4A1 Gene Mutation – Beyond a Vascular Syndrome,” Seizure‐European Journal of Epilepsy 1, no. 31 (2015): 19–21, 10.1016/j.seizure.2015.06.014. 26362372

[6] S. Lanfranconi and H. S. Markus , “COL4A1 Mutations as a Monogenic Cause of Cerebral Small Vessel Disease: A Systematic Review,” Stroke 41, no. 8 (2010): e513–e518. 20558831 10.1161/STROKEAHA.110.581918

[7] E. Cuadrado‐Godia , P. Dwivedi , S. Sharma , et al., “Cerebral Small Vessel Disease: A Review Focusing on Pathophysiology, Biomarkers, and Machine Learning Strategies,” Journal of Stroke Korean Stroke Society 20 (2018): 302–320. 30309226 10.5853/jos.2017.02922 PMC6186915

[8] D. S. Kuo , C. Labelle‐Dumais , and D. B. Gould , “COL4A1 and COL4A2 Mutations and Disease: Insights Into Pathogenic Mechanisms and Potential Therapeutic Targets,” Human Molecular Genetics 21, no. R1 (2012): R97–R110. 22914737 10.1093/hmg/dds346 PMC3459649

[9] J. K. Kim , H. W. Gabel , R. S. Kamath , et al., “Functional Genomic Analysis of RNA Interference in *C. elegans* ,” Science 308, no. 5725 (2005): 1164–1167. 15790806 10.1126/science.1109267

[10] L. S. De Vries , C. Koopman , F. Groenendaal , et al., “COL4A1 Mutation in Two Preterm Siblings With Antenatal Onset of Parenchymal Hemorrhage,” Annals of Neurology 65, no. 1 (2009): 12–18. 19194877 10.1002/ana.21525

[11] S. Shah , S. Ellard , R. Kneen , et al., “Childhood Presentation of COL4A1 Mutations,” Developmental Medicine and Child Neurology 54, no. 6 (2012): 569–574. 22574627 10.1111/j.1469-8749.2011.04198.x

[12] M. E. C. Meuwissen , D. J. J. Halley , L. S. Smit , et al., “The Expanding Phenotype of COL4A1 and COL4A2 Mutations: Clinical Data on 13 Newly Identified Families and a Review of the Literature,” Genetics in Medicine Nature Publishing Group 17 (2015): 843–853. 25719457 10.1038/gim.2014.210

[13] P. Maurice , L. Guilbaud , J. Garel , et al., “Prevalence of COL4A1 and COL4A2 Mutations in Severe Fetal Multifocal Hemorrhagic and/or Ischemic Cerebral Lesions,” Ultrasound in Obstetrics and Gynecology 57, no. 5 (2021): 783–789. 32515830 10.1002/uog.22106

[14] I. Sibon , I. Coupry , P. Menegon , et al., “COL4A1 Mutation in Axenfeld‐Rieger Anomaly With Leukoencephalopathy and Stroke,” Annals of Neurology 62, no. 2 (2007): 177–184. 17696175 10.1002/ana.21191

[15] S. Cao , Y. Li , W. Xu , and M. Xia , “Reader Response: Clinical Relevance of Acute Cerebral Microinfarcts in Vascular Cognitive Impairment,” Neurology Lippincott Williams and Wilkins 94 (2020): 329–330. 32066641 10.1212/WNL.0000000000008970

[16] S. Marini , C. D. Anderson , and J. Rosand , “Genetics of Cerebral Small Vessel Disease,” Stroke 51, no. 1 (2020): 12–20. 31752611 10.1161/STROKEAHA.119.024151 PMC7337039

[17] M. Mancuso , M. Arnold , A. Bersano , et al., “Monogenic Cerebral Small‐Vessel Diseases: Diagnosis and Therapy. Consensus Recommendations of the European Academy of Neurology,” European Journal of Neurology 27, no. 6 (2020): 909–927. 32196841 10.1111/ene.14183

